# The Medical Council and Unqualified Practice

**Published:** 1909-03-20

**Authors:** 


### The Hospital

A Journal of

The Medical Sciences and Hospital Administration.

New Series. No. 107, Vol. IV. [No. 1179, Vol. XLV.] SATURDAY, MARCH 20, 1909.

### THE MEDICAL COUNCIL AND UNQUALIFIED PRACTICE.

The freedom accorded to unqualified practitioners of medicine in these islands has long been a legitimate source of complaint. The complaint comes from those who have passed through a long and costly apprenticeship to fit them for a task which cannot be properly undertaken without it, yet is widely pursued by individuals whose only credentials are a highly-developed commercial instinct and an unblushing effrontery. Untrammelled by any considerations of self-respect or solicitude for professional honour, irregular practitioners take the field with a material advantage; and this, in view of the amazing credulity of the public in therapeutic matters, operates very hardly against those whose code of morals precludes resort to blatant advertisement and the broadcast distribution of promises incapable of fulfilment. It is worthy of notice, in passing, that this credulity is not, as one might expect, limited to the uneducated classes. It is almost equally apparent in the upper ranks of society, among those whose education has been of the most expensive and has included all the accomplishments of good-breeding, while omitting, it would seem, those of good sense.

But, as has been pointed out before now, it is often easier to grumble at a hardship than to find a remedy, and the difficulties in the way of suppressing unqualified practice are no slight ones, particularly since any professional move in this direction is sure to be interpreted as a mere expression of jealousy. Yet jealousy is not the right word to apply to an emotion based upon a sense of wrong, both public and private, like that under notice. The law has recognised the public benefit to be derived from legal qualification, and has given to qualified practitioners certain advantages which we are far from underrating. But these advantages are not lightly paid for; they are paid for in time—five years of study; they are paid for in cash, for a medical course costs much money; and they are paid for by the surrender of those commercial advantages which the quack exploits to such good financial purpose, and, since he lacks scruple, to the not inconsiderable detriment of the public.

With these facts in mind the General Medical Council appointed, in November, 1907, a committee to ascertain "what legal provisions exist in the Colonies and Dependencies of the Empire and in foreign countries for the prevention of medical practice by other than legally qualified persons, and to consider what steps should be taken to procure effective legislation for the same purpose in the United Kingdom of Great Britain and Ireland." In the following month the Registrar forwarded to the Privy Council a request that the various departments concerned should assist the Council in obtaining official information on the points under inquiry. The request was favourably entertained, and the Council in due time received authoritative statements touching the regulation of practice in all parts of the world except the United States, which did not respond to the invitation. The results have been tabulated and were issued with the report of the Committee in November last. In presenting its report the Committee assumes that every medical practitioner is aware of the injury done to the public by the practice of unqualified persons and companies which is carried on with impunity in this country. It believes that sufficient protection against such injury is not afforded to the public by the present Medical Act, that the injury is grave and increasing, and that a remedy must be found in legislation such as exists in most civilised countries. But it recognises, very wisely, that before remedial legislation can be looked for, it is necessary that the public should be made more fully aware of the dangerous abuses which exist under present conditions. This, it believes, can be best accomplished by the institution of a Governmental Commission of Inquiry.

Dr. Langley Brown's sub-committee has recommended in consequence that the Council should adopt the following resolution: "That the General Medical Council, being of opinion that the present Medical Acts do not sufficiently enable 'persons requiring medical aid to distinguish qualified from unqualified practitioners,' and that it is contrary to the interest of the public that medical and surgical practice should be carried on with impunity by persons holding no recognised qualifications, requests the Government to take steps for the appointment of a Royal Commission to inquire into the evil effects produced by the unrestricted practice of medicine and surgery by unqualified persons."

This resolution was adopted, and we must wait the event with patience. It is decidedly time that something more than grumbling was attempted, and we believe the Council has acted wisely in collecting data prior to any definitive move. It is an evidence of serious purpose and of confidence in the justice of the cause, and these things will not be lost upon the powers that be, nor upon those members of the general public whose respect is worthy of consideration.

